# The Microrna-143/145 Cluster in Tumors: A Matter of Where and When

**DOI:** 10.3390/cancers12030708

**Published:** 2020-03-17

**Authors:** Valeria Poli, Laura Seclì, Lidia Avalle

**Affiliations:** Molecular Biotechnology Center, Department of Molecular Biotechnology and Health Sciences, University of Turin, Via Nizza 52, 10126 Turin, Italy; laura.secli@unito.it

**Keywords:** miRNA-143, miRNA-145, tumorigenesis, EMT, oncogenes, oncosuppressors

## Abstract

The establishment and spreading of cancer involve the acquirement of many biological functions including resistance to apoptosis, enhanced proliferation and the ability to invade the surrounding tissue, extravasate from the primary site, survive in circulating blood, and finally extravasate and colonize distant organs giving origin to metastatic lesions, the major cause of cancer deaths. Dramatic changes in the expression of protein coding genes due to altered transcription factors activity or to epigenetic modifications orchestrate these events, intertwining with a microRNA regulatory network that is often disrupted in cancer cells. microRNAs-143 and -145 represent puzzling players of this game, with apparently contradictory functions. They were at first classified as tumor suppressive due to their frequently reduced levels in tumors, correlating with cell survival, proliferation, and migration. More recently, pro-oncogenic roles of these microRNAs have been described, challenging their simplistic definition as merely tumor-suppressive. Here we review their known activities in tumors, whether oncogenic or onco-suppressive, and highlight how their expression and functions are strongly dependent on their complex regulation downstream and upstream of cytokines and growth factors, on the cell type of expression and on the specific tumor stage.

## 1. Introduction

The process of tumorigenesis involves the dysregulation of many vital biological functions including cell proliferation, apoptosis, resistance to anoikis, acquirement of drug resistance features and, ultimately, of the ability to migrate, intravasate and extravasate at distant organ sites to give origin to metastases, which are the main cause of death for cancer patients [1]. A multitude of growth factors, hormones, protein-coding genes, and non-coding RNAs are involved in the fine regulation of these functions, which require coordinated activation and inactivation of specific genes. MicroRNAs (miR) are a class of conserved small non-coding RNAs (18–22 nucleotides), that function via base-pairing complementarity of their seed sequences (nucleotides 2–7) typically with the 3’UTR of target RNA, leading to inhibition of the target mRNA translation and often to its degradation [2]. MicroRNAs have important functions in finely regulating the expression of about 60% of the genes in mammals [3], and they have emerged as key players in cancer by targeting sets of functionally correlated genes, exerting both pro- and anti-oncogenic roles [4] acting both directly on tumor cells and on the pro-oncogenic/anti-oncogenic functions of stromal cells [5].

**Figure 1:**
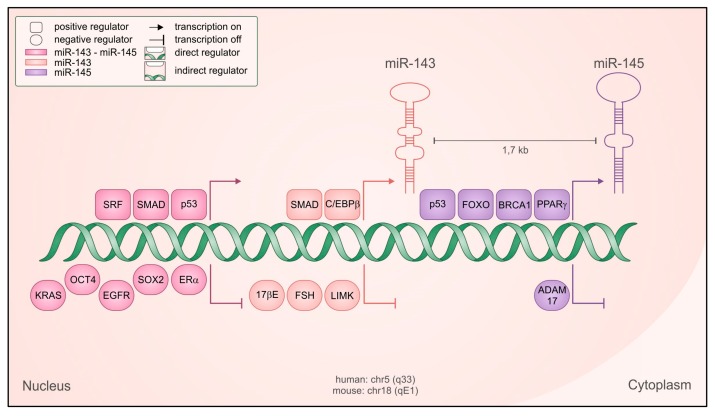
Regulation of miR-143 and -145 transcription. Schematic representation of the genetic locus containing the microRNAs, with both positive (squared) and negative (rounded) regulators shown to be relevant for their transcription, in both normal and cancer cells. Regulators with direct transcriptional activity are depicted closer to the DNA, and the color code indicates if they regulate both microRNAs as a cluster (fuchsia) miR-143 only (pink), miR-145 only (violet).

Among these, microRNA-143 and microRNA-145 have been shown to be often dysregulated in tumors (recently reviewed in reference [6]). These two evolutionarily conserved microRNAs are transcribed as a cluster from chromosome 5 in humans (5q33) and chromosome 18 in the mouse (18qE1), with their transcriptional units located at approximately 1.4 kb of distance. The two primary transcripts described are a bicistronic 11 kb RNA and a 1.9 kb smaller transcript containing miR-145 only [7,8]. Accordingly, their expression is mostly coordinated but miR-145 can also be transcribed independently under the positive control of tumor protein p53 (p53) [9], opposed to POU class 5 homeobox 1 (Oct4)-mediated repression [10] (Figure 1). The common regulatory elements lie in the 4.5 kb region upstream of miR-143, where binding sites for the serum response factor (SRF)/Myocd [7,11,12] and SMAD family member (SMAD) transcription factors [12] have been identified and characterized. Accordingly, SMAD-dependent transcriptional activation by transforming growth factor beta (TGF-β) plays a prominent role in controlling the expression of the cluster [12]. Despite related biological functions, these two microRNAs do not share sequence similarity and mostly recognize different sets of target genes [7,11,12,13], although they can synergistically regulate a subset of shared targets [14].

In human normal tissue a high expression of miR-143 and -145 has been described in cervix and colon, followed by prostate, uterus, small intestine and stomach, while very low expression was detected in kidney, placenta, testis, spleen, skeletal muscle, liver, and brain [8]. Both microRNAs have been characterized as key players in the differentiation of vascular smooth muscle cells (VSMC) during development [10,11,13]. Indeed, expression of the miR-143/145 cluster in adult mice was initially described as confined to visceral and vascular smooth muscle cells [7,11,13], and their activity can alone induce the differentiation of multipotent neural crest stem cells into VSMCs, a well-known TGF-β-dependent form of EMT [11,15]. Vascular injury normally leads to dedifferentiation of SMCs, which then proliferate and migrate to the injury site giving origin to the neointima scar tissue that ultimately causes vascular obstruction [16]. This phenomenon requires miR-143 and -145 activity, since genetic inactivation of the miR-143/145 cluster in adult tissues leads to impaired neointima formation upon vascular injury [7,13], correlating with disorganization of actin stress fibers and reduced migratory ability of miR-143 and -145-null SMCs [7]. Accordingly, both microRNAs functionally converge on the regulation of a significant number of common target genes ultimately modulating the activity of SRF and actin dynamics, and miR-143/145-null mice display arterial hypotension [7]. In the same vein, interference with miR-145 expression in zebrafish affects the contractility of intestinal SMCs, with consequent defects in gut peristalsis and swim bladder inflation [17].

Down-regulation of both miR-143 and -145 levels has frequently been observed in epithelial cancers and in B-cell malignancies [18]. Moreover, the locus is located in the 5q33 fragile site of human chromosome 5 that is often deleted in cancer [19], and ectopic expression of both microRNAs was reported to inhibit proliferation, induce apoptosis, and/or suppress anchorage-independent growth, epithelial to mesenchymal transition (EMT) and tumor-forming ability of different cancer cell types both in vitro and in vivo [6,16,17,18,19]. These evidences have led to consider both microRNAs as oncosuppressors, as recently reviewed by Das and Pillai [6]. Several reports later challenged this concept for both miR-143 and miR-145, showing that their expression correlated with invasion, disease grade and progression in specific types of tumors [20,21,22,23]. Thus, their role in cancer may be more complex than previously believed and possibly also depend on the cell type(s) of expression and on tumor stage, similar to what described for their inducer TGF-β [24]. The ability to enhance tumor invasion and progression is in keeping with their prominent role in promoting migration and contractility of vascular and visceral smooth muscle cells [7,17] and to induce epithelial wound healing with a paracrine mechanism [25]. Similar activities could be involved in supporting the pro-tumorigenic functions of cancer associated fibroblasts (CAFs), which have features of activated myofibroblasts, and in enhancing migration and invasion of epithelial tumor cells undergoing EMT. Here, we will review the main findings supporting the oncogenic versus the oncosuppressor roles of the two microRNAs, alone or in combination as a cluster, and discuss possible explanations for the contrasting results reported.

## 2. Expression in Tumors

As mentioned above, miR-143 and -145 have been found to be both down-regulated and up-regulated in tumors of different origins, suggesting that their role may vary according to tumor type and stage. Below we summarize the main findings in both directions with emphasis on their up-regulation, which has never been reviewed yet.

### 2.1. Downregulation of miR-143/miR-145 in Tumors

As recently reviewed by Das and Pillai [6], the downregulation of miR-143 and -145 was described in a considerable number of cancer cell lines and tumors, including those of the colon [26,27], prostate [28], ovary [29,30], bladder [31,32], breast [33,34], renal cell carcinoma [35], neuroblastoma [36], and osteosarcoma [37]. Moreover, lower expression rates of both microRNAs were reported to correlate with shorter disease-free survival and the presence of metastases in prostate, cervical and breast cancer, hepatocellular carcinoma (HCC) and glioma [6]. Human breast cancer cell lines were shown to express reduced levels of miR-143 and -145 compared to the MCF10A immortalized normal epithelial cells and to normal tissue, and miR-145 but not miR-143 overexpression inhibited proliferation of several breast cancer cell lines [38].

In partial contrast, although the expression of the cluster in bladder cancer cells is generally lower than in the adjacent non-tumor tissue, high-grade muscle-invasive bladder tumors display higher expression of both microRNAs when compared to non-invasive or low grade samples, correlating with poor overall survival [39]. These data support previous evidence suggesting up-regulation of miR-143 and -145 expression correlating with poor survival in both invasive and non-invasive bladder cancer [40,41].

How are miR-143 and -145 downregulated in tumors? While in myelodysplastic syndrome miR-143 and -145 downregulation is easily explained by genetic deletion, since the locus is located in the region of the often deleted 5q32 fragile site [19], the mechanisms are less clear in other tumor types. Genetic loss has also been detected in ovarian carcinomas [42], while epigenetic silencing via CpG island methylation of the miR-145 promoter was demonstrated in different tumors [43,44,45]. The transcriptional regulators peroxisome proliferator activated receptor gamma (PPARγ), CCAAT enhancer binding protein beta (C/EBPβ), forkhead box O (FoxO), BRCA1 DNA repair associated (BRCA1), and p53 have all been reported to positively regulate transcription of the cluster or of miR-145 alone [6] (Figure 1). Some of them are well known tumor suppressors whose downregulation in cancer may indirectly result in reduced miR-143 and -145 expression. Several oncogenic signals can inhibit the expression of the cluster, including the epidermal growth factor (EGF)/ epidermal growth factor receptor (EGFR) [46] signaling pathway and the oncogene KRAS, alone or in combination with MAPK or PI3K family members and acting via the Ras-responsive Element Binding Factor (REBB1) [47]. Additionally, follicle stimulating hormone (FSH), cortisol and 17-Beta-estradiol or its ERa receptor, often upregulated in cancer, were reported to repress the expression of both microRNAs in gastric, breast and ovarian cancer [6]. Interestingly, some of the factors shown to be involved in microRNAs repression are at the same time targets of either miR-143 (*LIMK1, SOX2*) or miR-145 (*ADAM17, NEDD9, SOX2*) [6] (Figure 1), potentially triggering a double negative feedback loop. 

### 2.2. Upregulation of miR-143/miR-145 in Tumors

Since 2007, discordant evidence started to emerge challenging the concept of miR143/145 microRNAs as solely onco-suppressive. In particular, their expression has often been correlated with enhanced motility, invasion, and metastasis, as outlined below and summarized in Table 1. Szafranska and colleagues reported overexpression of both microRNAs in samples from pancreatic ductal adenocarcinomas and pancreatitis, although their levels were below detection in 6 pancreatic cancer cell lines [48]. miR-143 levels were shown to positively correlate with metastases in Hepatitis B-related hepatocellular carcinoma patients, and its blockade was sufficient to inhibit metastasis in a transgenic mouse model of hepatic cancer [21]. In this vein, Fan and co-authors reported that the enhanced aggressiveness acquired by sphere-derived prostate cancer stem cells upon growth in adhesion functionally correlated with strongly increased miR-143 expression [49], the inhibition of which impaired their migration and invasion via increased expression of the tumor suppressor *FIBRONECTIN TYPE III DOMAIN CONTAINING 3B (FNDC3B)*, a microRNA target. We have recently demonstrated that miRs-143 and -145 are strongly overexpressed in the highly aggressive mouse mammary tumors that develop in mice expressing the NeuT oncogene together with constitutively active STAT3 [50,51]. High expression was required for the disrupted cell junction phenotype displayed by derived NeuT-STAT3C cell lines, which could be normalized by silencing either microRNA. Inhibition of miR-143 significantly reduced in vivo extravasation of the NeuT-STAT3C cells, suggesting a key role in enhancing colonization at distant organs [50]. Moreover, overexpressing either microRNA in the normal murine mammary gland (NMuMG) cells increased cell motility, inducing the expression of several EMT markers, of TGF-β targets and of junction proteins. The mechanism involved enhanced basal SMAD-mediated transcription, suggesting a positive feedback loop between miR-143 and the TGF-β pathway. Of note, a negative feedback loop with TGF-β has instead been proposed in hematopoietic stem cells (HSC). Mice lacking miR-143 and -145 displayed decreased long-term HSC numbers, predisposition to myeloproliferative activity and increased bone marrow expression of the common target gene an adaptor that facilitates the activation of SMADs, and this phenotype could be recapitulated by transplantation with DAB adaptor protein 2 (DAB2)-overexpressing HSCs into wild type mice [52]. Interestingly, increased DAB2 expression was also detected in HSCs from myelodysplastic syndrome patients presenting a deletion of the locus [52].

miR-145 expression is upregulated in primary colorectal cancer with lymph node metastasis. Indeed, although both metastatic and non-metastatic samples displayed miR-145 downregulation as compared to the normal tissue, its expression was significantly higher in the samples derived from metastatic patients. miR-145 overexpression promoted CRC cell migration in vitro and enhanced metastasis in vivo, with no effect on cell proliferation. The effect on cell motility was brought about by miR-145-mediated stabilization of heat shock protein (HSP) 27 [22]. In agreement with the idea that the specific role of tumor players may change according to disease stage, miR-145 (but not miR-143) was shown to promote EMT and increased anchorage-independent growth when overexpressed in a metastatic CRC cell line [53], and to be induced by neoadjuvant therapy in esophageal adenocarcinoma (EAC), where its levels correlated with a decreased disease-free survival [56]. Interestingly, miR-145 overexpression could lead to opposite effects in different types of esophageal cancer. miR-145 levels are normally downregulated in esophageal squamous cell carcinoma (ESCC), where its overexpression can inhibit cell proliferation and invasion [57,58]. In contrast, in esophageal adenocarcinoma (EAC) high miR-145 expression levels correlate with poor prognosis, enhancing cell invasion, adhesion to fibronectin and resistance to anoikis [20]. Finally, glioblastoma cells selected to become more invasive and aggressive display greatly increased levels of both miR-143 and -145 without reduced cell viability [54], and miR-145 inhibition reduces their in vitro invasion [54]. These effects were attributed to miR-145-mediated downregulation of SLIT-ROBO RHO GTPase activating protein 1, which carries miR-145 seeds [59].

**Figure 2:**
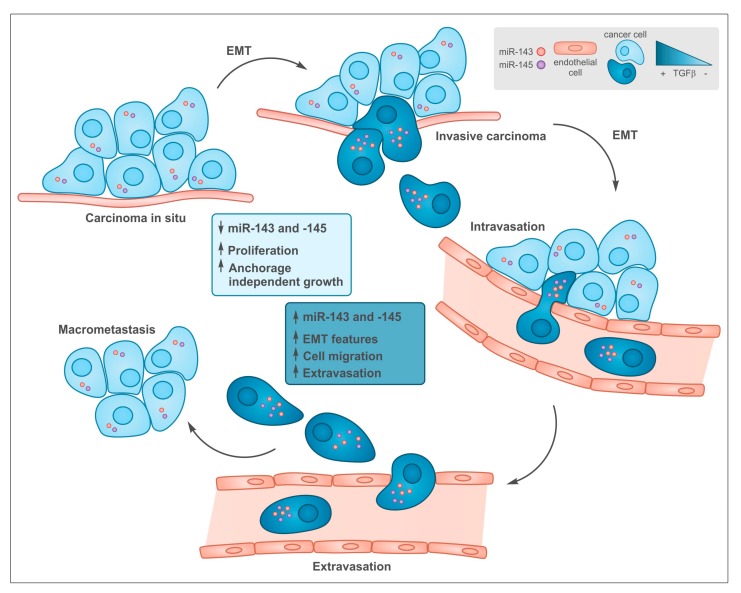
Common functions of transforming growth factor beta (TGF-β) and miR-143 and -145 during tumor progression. In early stages low levels of both TGF-β and miR-143/145 are required to allow cell proliferation at the primary sites. During tumor progression, some cells undergo epithelial to mesenchymal transition (EMT), which is induced by TGF-β that, among many other targets, triggers the up-regulation of miR-143 and -145 expression, which in turn, support tissue invasion and metastasis formation.

How is the cluster up-regulated in tumors? One of the best characterized inducers of the miR-143/-145 cluster that is often induced during tumorigenesis is TGF-β, acting via SMAD factors both at the transcriptional and post-transcriptional level [12,50,60]. TGF-β has a well-known dual role in cancer [15,61], initially suppressing tumorigenesis via its anti-proliferative actions, while later driving metastasis thanks to its strong ability to induce epithelial to mesenchymal transition (EMT), cell motility and invasion [61,62]. This is well in line with the differential effects exerted by both microRNAs in late-stage, aggressive tumors vs early tumors with a good-prognosis (Figure 2). Another transcription factor often up-regulated in tumors and able to activate miR-143 is Liver-enriched Activator Protein LAP, the full-length C/EBPβ isoform, which is known to promote pro-survival pathways upon ER stress correlating with enhanced drug resistance of triple negative breast cancer [63].

Finally, several functional single nucleotide polymorphisms (SNPs) have been identified in the regulatory regions of the two microRNAs, with most of the tested variants correlating with lower transcriptional activity and increased tumor risk. Li and co-authors first described several SNPs in the regulatory region of the miR-143/145 cluster, several positively correlating with CRC development [64]. The rs353292 variant, which displays reduced transcriptional activity in a Luciferase reporter assay [65], was shown to correlate with lower miR-143, but not miR-145, expression levels and CRC development in tumor samples. Similar findings were reported in the context of the rs4705342T > C and the rs4705343T > C variants, with the T alleles displaying reduced promoter activity and increased prostate cancer or cervical squamous cell carcinoma development [66,67]. A few SNPs correlating with reduced transcription and lower cancer risk have also been described, as in the case of the rs353293 allele in the cluster’s promoter, which dictates lower transcriptional activity of a luciferase reporter and was a protective genetic marker in bladder cancer [68].

## 3. Potential Mechanisms Mediating microRNAs Functions in Tumors

Although anti-proliferative effects of miR-143 and -145 have been described, both microRNAs have mainly been involved in the control of multiple aspects related to the metastatic process via the regulation of EMT, which is believed to play a key role in the ability of tumor epithelial cells to acquire mesenchymal features that enable them to extravasate, survive in the circulation, intravasate at distant organs and grow to give origin to metastatic lesions [69,70] (Figure 2). Below we review the main data describing the role of miR-143 and -145 in regulating the EMT and EMT-related processes (Section 3.1), and the known target genes that could mediate their functions (Section 3.2).

### 3.1. miR-143 and -145 and EMT

Indeed, many studies have investigated the ability of the two microRNAs to regulate cell movement and adhesion in the context of EMT and metastasis formation. Despite them being well-known transcriptional targets of the EMT driver TGF-β [12], and despite their physiological role in the induction of EMT-like phenomena such as the differentiation of contractile and migrating SMCs, the majority of studies in tumors report a repressive role in EMT.

The expression of both miR-143 and miR-145 is reduced in esophageal squamous cell carcinoma clinical samples [71,72], where their overexpression was shown to downregulate mesenchymal markers, reducing cell migration and metastatic ability [71,72]. miR-145 ectopic expression inhibited cell migration by reducing the levels of FASCIN1 (FSCN1), an actin bundling protein involved in cytoskeletal dynamics, and regulating several EMT features in several breast cancer cell lines [73]. The upregulation of miR-143 in a similar context increased E-CADHERIN mediated cell–cell adhesion while reducing the expression of mesenchymal markers and cell migration [74].

Several components of the TGF-β pathway itself have been proposed to be targeted by miR-143 and -145, suggesting that they may take part in a negative feedback loop. miR-145 levels are reduced in tumor samples and cell lines with respect to normal tissue in non-small cell lung cancer and inversely correlated with those of SMAD3, one of the key transcription factors involved in TGF-β responses and later characterized as a direct miR-145 target [75]. Indeed, overexpression of miR-145 was sufficient to reduce TGF-β-induced EMT in this cell model. Similarly, in a model of nasopharyngeal cancer miR-145 is downregulated, correlating with high SMAD3 levels and increased cell migration [76]. ACTIVIN A, a cytokine of the TGF-β superfamily that contributes to SMAD2 and SMAD3 activation, is targeted by miR-143 and miR-145 in a model of oral squamous cell carcinoma [77]. In patient samples, low microRNAs expression correlates with high ACTIVIN A levels and poor prognosis. As already described in Section 2.2, moreover, both microRNAs can target the TGF-β facilitator *DAB2* in bone marrow stem cells [52]. 

The transcription factors zinc finger E-box homeobox 1 (ZEB1) and 2 (ZEB2) are involved in EMT induction by repressing epithelial genes such as *E-CADHERIN, MUCIN* (*MUC*) *1*, *PLAKOPHILIN 2, CONNEXIN 26*, TIGHT JUNCTION PROTEIN 3 (*ZO-3*) [78]. Both are targeted by the miR-200 family, which is often downregulated in tumor EMT [79] resulting in their up-regulation [80]. In prostate cancer, ZEB2 was shown to be involved in a double negative feedback loop with miR-145 [81], and indeed ZEB2 and miR-145 levels are negatively correlated in prostate cancers. Moreover, miR-145 overexpression in DU145 prostate cancer cells could downregulate the mesenchymal markers N-CADHERIN, VIMENTIN and FIBRONECTIN while increasing E-CADHERIN levels [81,82]. Similar results were obtained in PC3 prostate cancer cells by Peng and colleagues, who showed that miR-143 and -145 levels were lower in samples from bone metastatic lesions that in primary prostate cancers, suggesting that their downregulation might help cells to undergo EMT and escape from the primary site [83]. *ZEB2* emerged as a target of miR-145 also in cervical cancer cells, resulting in a reduction in VIMENTIN and SNAIL, upregulation of E-CADHERIN and reduced migration and invasion [84]. Interestingly, Lei and co-authors reported that the long non-coding (lnc) RNA *TUG1*, often overexpressed in papillary thyroid and bladder cancer, can act as a competing endogenous RNA for miR-145, resulting in the induction of its targets ZEB1 and ZEB2 and enhanced EMT [85,86]. Also the lncRNA *UCA1* was shown to induce EMT by repressing miR-145 expression, leading to increased levels of ZEB1, ZEB2, and FSCN1 [87].

Paradoxically, most data supporting pro-tumorigenic functions of the two microRNAs also correlate their activity with enhanced cell movement and metastasis, although not always specifically investigating EMT. Fan and co-authors reported a direct correlation between in vitro and in vivo prostate cancer cells motility and the expression levels of miR-143, whose inhibition reduced migration and invasion [49]. miR-145 overexpression promoted CRC cell migration, EMT, enhanced anchorage-independent growth in vitro [53] and metastasis in vivo, with no effect on cell proliferation [22]. Increased cell invasion, adhesion to fibronectin and resistance to anoikis were observed in Esophageal Adenocarcinoma [20]. On the other hand, miR-145 inhibition limited in vitro glioblastoma cells invasion [55]. In the context of non-small cell lung cancer, Yang and colleagues showed that TGF-β- induced miR-145 reduced the expression of miR-20a by targeting c-myc, resulting in enhanced expression of its target transforming growth factor beta receptor II TGFBRII and sensitizing cells to TGF-β treatment [88]. Similarly, interference with miR-143 in aggressive mouse mammary tumor cells diminished their motility and in vivo extravasation, correlating with increased SNAIL and ZO1 expression [50]. Accordingly, miR-143 or -145 overexpression in NMuMG mouse mammary epithelial cells increased their motility [50], correlating with enhanced activity of a SMAD-reporter gene, increased expression of the EMT markers Snail and N-cadherin and downregulation of the epithelial markers E-CADHERIN, ZO1, ZO3, and OCCLUDIN. Transcriptomic analysis revealed significantly overlapping patterns of down-regulated genes, including several transcription factors involved in regulating the *ZO1, ZO3,* and *OCCLUDIN* promoters. Moreover, downregulation of the putative miR-143/145 common target *MEKK2* could explain the observed decreased activation of ERK/MEK kinases, which are known to stabilize TGIF, a SMAD transcriptional co-repressor whose protein levels are reduced in the overexpressing cells. In turn, reduced TGIF levels may trigger the observed increased activity of the TGF-β pathway [50].

### 3.2. Proposed and Validated Targets of the microRNAs

This host of observations suggests that miR-143 and miR-145 role in cancer may vary according to cell type and tumor stage, likely due at least in part to the differential repertoire of target genes in different cell types both of tumor and stromal origin, and at different stages of tumor progression. The shifting balance and abundance of targets, varying the competing endogenous RNA milieu, may influence the susceptibility of specific mRNAs [89]. Additionally, the expression of miR-143 and -145 is regulated by cytokines, hormones and growth factors whose relative abundance vary during tumor progression [6], as described for TGF-β in session 3. It is therefore not surprising that contrasting data have also been published concerning the role of the two microRNAs in cell movement and EMT, as outlined in the previous section.

Despite the observation that the two microRNAs share a number of targets, suggesting at least partially converging functions, they do not show any sequence similarity and mostly recognize different sets of target genes, some of which exhibit oncogenic properties. miR-145 was shown to directly target oncogenic/stem cells factors such as *C-MYC* (downstream of *P53* [9,90]),KRUPPEL LIKE FACTOR 4 (*KLF4), SRY-BOX TRANSCRIPTION FACTOR 2 (SOX2)*, and *OCT4* [10]. The latter is also a miR-145 transcriptional inducer, suggesting an OCT4-miR-145 negative feedback loop regulating the balance between pluripotency and differentiation. Moreover, genes involved in cell death, proliferation, cell cycle, gene expression and cancer have been described as miR-145 targets, including the non-receptor Tyrosine Kinase *YES1* and the transcription factor *STAT1* [91]. miR-143 and 145 share tumor growth-related target RNAs such as the oncogene *KRAS* and *RREB1* (Ras-responsive element-binding protein) [92,93,94,95]. As to miR-143, it was shown to target the mitogen-activated protein kinase 7 (*ERK5)* [96,97,98,99,100], contributing to the inhibition of prostate cancer progression in mice [96]. More recently, the TGF-β activated kinase 1 (*TAK1)*, involved in controlling the activation of p38 MAPK, has also been reported as a miR-143 target in human ovarian cancer cells [101]. Finally, proteins involved in cell–cell adhesion and cell motility can be targeted by both microRNAs. For example, the cell–cell adhesion protein *JAM-A* [102] and the actin bundling protein *FSCN1* [55,72,102,103,104,105] have both been described as miR-145 targets, suggesting inhibition of cell adhesion and movement. Indeed, overexpression of miR-145 in breast cancer cell lines decreased actin stress fibers, and miR-143 was shown to regulate ADDUCIN 3 [106] and FIBRONECTIN [21,49], leading to reduced cell migration. In contrast, NF-ΚB-induced miR-143 expression, leading to FIBRONECTIN downregulation, was linked to enhanced cell motility and metastasis in HBV-induced hepatocellular carcinomas [45] and in a prostate cancer model [46]. Negative feedback loops have also been detected between genes whose expression is often dysregulated in cancer, which are both targets of the microRNAs and their negative regulators, as in the case of ADAM17 and NEDD9 with miR-145 in RCC and glioma cells, respectively [107,108], of Sox2, which downregulates the expression of the cluster [109], and of LimK1, a negative regulator of miR-143 in Non-Small Cells Lung Cancer (NSCLC) [100].

Recently, SILAC proteome combined with microarray analyses were performed in a colon cancer cell line upon re-expression of miR-143 and -145 at levels comparable to normal colon mucosa [14], allowing to identify downregulated proteins, 21 of which are shared targets [14]. Among these, CADHERIN (CDH) 17, FSCN1 and MOUSE DOUBLE MINUTE (MDM) 2 play important roles in tumor progression. In contrast, miR-143 or miR-145 overexpression in a human CRC cell line led to the downregulation of many components of apoptotic processes (i.e., SOD1, PRDX2 and PRDX6 for miR-143, and heat shock proteins, glutathione S-transferases and calreticulin for miR-145 [110]), suggesting miR-143-mediated cell survival. 

## 4. When the “Where” Matters: Tissue Specific Expression of microRNAs-143 and -145

**Figure 3:**
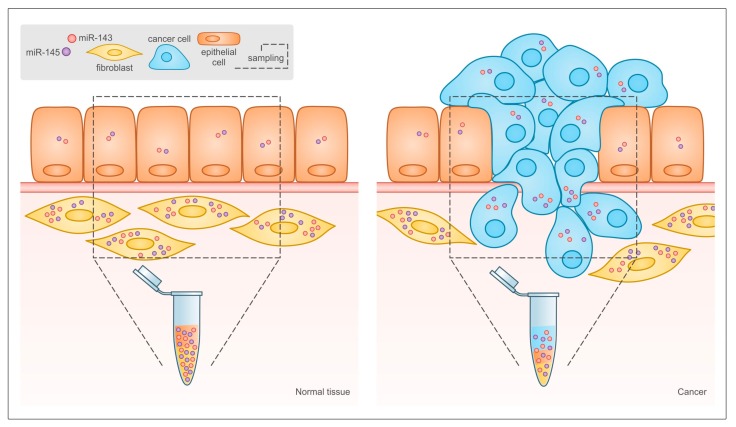
Relative abundance of different cell types in normal tissue and tumor may lead to misinterpretation of miR-143 and -145 expression data in solid tumors. In traditional bioptic samples, the amount of miR-143 and -145 that can be measured by RT-PCR is the result of their expression levels in the bulk of the tumor, including both cancer and stromal cells. As shown here, the expression of both microRNAs is much higher in stromal than in epithelial cells in normal tissues, and the relative abundance of the two cell types is inverted in tumors. This leads to reduced microRNA levels in the tumor bulk even when their expression is actually increased in tumor cells, and to the misleading conclusion that miR-143 and miR-145 are always down-regulated in tumors.

Emerging evidence underlines the functional relevance of the cellular context where the two microRNAs are expressed. In particular, Kent and colleagues reported that the downregulation of miRs-143 and -145 in cancer observed in many studies may not take into account the fact that bulk tumor tissue and not isolated cancer cells were analyzed. Indeed, in the normal colon both microRNAs are highly expressed in mesenchymal stromal cells like fibroblasts and smooth muscle cells and at very low levels in epithelial cells [111], while the ratio between stromal and tumor tissue is completely inverted in colonic tumors, as illustrated in Figure 3. This idea was confirmed by mouse studies, which showed that in the intestine miRs-143 and -145 are expressed almost exclusively by mesenchymal cells, mainly smooth muscle cells and myofibroblasts, and are not detectable in the epithelial cells [25], in line with earlier studies [7,112]. Chivukula and colleagues demonstrated that, although miR-143 and -145 null mice did not display any defect in intestinal development, adult animals showed severely defective intestinal regeneration. Interestingly, the same phenotype could be reproduced by specific mesenchymal deletion of the microRNAs. Thus, miRs-143 and -145 play a crucial role in epithelial homeostasis and tissue repair in the intestine by regulating the functions of mesenchymal cells, in keeping with their originally described ability to stimulate VSMCs differentiation during blood vessel injury [7,11,112,113]. More recent data along this line show that TGF-β-induced miR-145 enhances myofibroblasts differentiation in both the lung and the skin, leading when excessive to lung fibrosis and hypertrophic scarring after wound healing [114,115].

In the context of cancer, it was shown that normal lung epithelium does not express either miR-143 or miR-145, and indeed their tumor-specific depletion did not enhance the development of lung adenocarcinoma in a genetic mouse model. In contrast, lung endothelial cells express high level of both microRNAs, which dramatically promote neo-angiogenesis and tumor formation [116,117]. In situ hybridization experiments on NSCLC patient samples showed higher miR-143 and -145 expression in both epithelial and stromal cells [118] from the tumor with respect to non-malignant lung tissue. In prostate cancer, in which miR-143 has been considered a tumor suppressor, recent microdissection studies reported the detection of very few copies of miR-143 in normal prostate epithelial cells as opposed to a very high expression in stromal cells [119]. In tumor specimens, stromal but not epithelial miR-143 expression decreased by 5-fold. Also in breast cancer tissue, where microarray analysis showed downregulated expression of both microRNAs as compared to normal breast tissue, correlating with bad prognosis, in situ hybridization on the same samples allowed the detection of high microRNAs expression in stromal cells. Finally, miR-143 was shown to be highly expressed in the fibroblasts, but not in the tumor cells of aggressive scirrhous type gastric cancer, correlating with bad prognosis [120]. In keeping with previous data [50,52,88], here miR-143 could positively regulate TGF-β signaling, increasing SMAD2 phosphorylation and resulting in enhanced expression of collagen type III [120]. 

All of the above observations strongly support the idea put forward by Kent and co-authors [111], and call for a re- interpretation of earlier results. New expression data should be derived from micro-dissected tissue or by in situ hybridization to avoid biases due to the differential cell composition of normal and cancer tissues (Figure 3).

## 5. Conclusions

The data discussed above clearly show that miR-143 and -145 can exert both pro-oncogenic and anti-oncogenic functions, depending on a number of factors. First, the balance between the many different factors and signals that can positively or negatively regulate their as well as their targets expression in different cell types. Second, their cell-specific functions, which in turn may stem from their cell-specific expression and availability of their target genes. Indeed, the phenotypic consequences of their over-expression or silencing dramatically vary according to the cell type, the transformed status and even the stage of the disease, suggesting that these microRNAs are neither truly oncosuppressors or oncogenes, but rather they can both promote and inhibit tumorigenesis acting at several levels and in different cell types. More precise analysis of all tumor cell components and cell-specific overexpression or interference experiments are needed to shed light on the mechanisms dictating miR-143 and -145 dichotomic functions, for which animal models inactivating the microRNAs locus in different cell types would be of paramount importance. At this stage, this analysis discourages the proposed attempts at clinical intervention based on manipulating their expression/activity [6].

## Figures and Tables

**Table 1:** Pro-tumorigenic functions of microRNA-143/145 cluster.

MiR	Target Gene	Type of Cancer	Cancer-Related Function	Reference
miR-143, miR-145	n.d.	Pancreatic ductal adenocarcinoma	Reduced in normal tissue compared to tumor, not detected in tumor cell lines	[48]
miR-143	*FIBRONECTIN TYPE III DOMAIN CONTAINING 3B (FNDC3B)*	Hepatitis B related hepatocellular carcinoma (HBV-HCC)	Upregulated by NF-KB, promotes cell migration, invasion and tumor metastasis	[21]
miR-143	*FNDC3B*	Prostate cancer	Upregulated during cancer stem cell differentiation, promotes metastasis	[49]
mir-145	*E-CADHERIN*	Colorectal cancer	When overexpressed in a metastatic cell line, it increases cell growth and leads to mesenchymal-like cell morphology	[53]
miR-145	n.d.	Esophageal adenocarcinoma	Do not affects cell proliferation, accelerates wound closure, enhances cell invasion, protects from anoikis	[20]
miR-145	stabilizes HSP27	Colorectal cancer	Associate with lymph node metastasis. When overexpressed in a CRC cell line, do not affects cell proliferation, promotes migration and invasion in vitro and in vivo	[22]
miR-143, miR-145	n.d.	Glioblastoma	Highly expressed in typical areas of invasion and in highly invasive GBM subpopulations, when downregulated invasion is reduced	[54]
miR-145	*srGAP1*	Glioblastoma	Enhances invasiveness	[55]
miR-143, miR-145	Proposed: *CREB1, MEKK2*	Mammary tumor	Induces EMT features, enhances cell migration	[50]
